# New drugs and new concerns: Gaining insight through Pharmacovigilance of direct acting Anti-Viral’s in chronic HCV patients

**DOI:** 10.12669/pjms.37.2.3400

**Published:** 2021

**Authors:** Zahid Yasin Hashmi, Muhammad Qasim Zia, Akram Bajwa, Maqsood Ahmed, Naveed Anwer, Mahwish Raza, Jaffer Bin Baqar

**Affiliations:** 1 Zahid Yasin Hashmi, FCPS Liver Center Faisalabad, Pakistan; 2 Muhammad Qasim Zia, FCPS Allama Iqbal Memorial Teaching Hospital, Sialkot, Pakistan; 3 Akram Bajwa, Ph.D. Liaquat University of Medical & Health Sciences, Hyderabad, Pakistan; 4 Maqsood Ahmed, FCPS Allied Hospital Faisalabad, Pakistan; 5 Naveed Anwer, MRCP Rehman Medical Institute Peshawar, Pakistan; 6 Mahwish Raza, Pharm-D Shaheed Zulfikar Ali Bhutto Institute of Science and Technology, Karachi, Pakistan; 7 Jaffer Bin Baqar, MSc. University of Karachi, Karachi, Pakistan

**Keywords:** Adverse Events, Chronic Hepatitis, Direct-Acting Antivirals

## Abstract

**Objective:**

The study aimed to assess the safety profile of Direct Acting Anti-Viral’s (DAAs) among patients with chronic Hepatitis C Virus (HCV).

**Methods:**

This multicenter, analytical cross-sectional study was conducted in six gastroenterology and Hepatology centers including Liver Center Faisalabad, Allama Iqbal Medical Institute and Liver Center DHQ Hospital Sialkot, Isra Hospital Hyderabad, Allied Hospital Faisalabad and Rehman Medical Institute Peshawar, between May 2018 and May 2019. The data regarding patient demographics, treatment plan and the frequency of Adverse Events (AEs), and their severity was collected using a pre-designed questionnaire and analyzed through SPSS version 20.0.

**Results:**

A total of 511 HCV patients were enrolled, with an overall male majority. Around 66.3% patients experienced a total of 419 AEs, out of which 61 events were suspected from DAAs while remaining 317 events were associated with Ribavirin. Pyrexia (24.6%) and fatigue (14.8%) were the most commonly reported AEs among patients receiving DAAs. Factors such as Ribavirin-based treatments and the presence of Cirrhosis were more likely to promote AEs occurrence OR [95%CI] i.e. 5.2(2.3-9.1) and 1.9(1.1-3.1) respectively (p < 0.05).

**Conclusion:**

It is concluded from the study results that DAAs have displayed promising outcomes due to the minimal and minor AEs reported.

## INTRODUCTION

The global burden of HCV infection is enormous mainly due to increased prevalence in the low and middle-income countries. The infection rate is estimated to be 2.3% in the Eastern Mediterranean Region as per World Health Organization (WHO).^1^ Whereas globally it affects more than 71 million people.^2^ The precise burden of HCV in Pakistan is not known, however, as per careful estimates, over 7.8 million Pakistani population is affected by HCV and the seropositivity of the virus is still increasing.^3^ Unfortunately the interlinked high burden of unsterile needle use, blood transfusion in the absence of optimal donor blood screening practices, dental procedure and other surgeries performed by unqualified practitioners, infrequent sterilization and intravenous (IV) drug abuse attributes to this high burden of HCV infection in the Pakistani population. 4

The therapeutic approach in relation to chronic HCV has always been challenging. Since 2011, DAAs have been approved for the management of HCV patients by the Food and Drug Administration (FDA) and European Medicines Agency (EMA) after conventional interferon.^5^ DAAs brought a revolution in the treatment of HCV and showed better compliance in terms of shorter duration of therapy, and a lesser number of adverse drug reactions reported as compared to Ribavirin and Interferon therapy.^6^ These DAAs have shown to achieve cure rates of over 90% regardless of previous treatment response, age, gender and ethnicity and are now part of preferred HCV treatment regimens as per international guidelines.^1^

Given this premise, now the next step in improving the HCV treatment protocol with DAAs is pharmacovigilance of the new drugs in order to improve the knowledge of drug safety. Different combinations of DAAs have been used in different groups of HCV patients worldwide and overall a lesser number of AEs are reported. However, now that the DAAs are prescribed increasingly around the globe, still few reports are available on the safety and occurrence of AEs based pharmacovigilance studies. Therefore, assessment of efficacy and safety through post-marketing studies is pivotal to make sure that DAAs are well tolerated with a better safety profile. Since much of the large data-based studies are conducted by high-income countries. Therefore, local evidence from Pakistan is scarce, we aim to report the safety profile of DAAs through this large multicentric study. The occurrence and spectrum of AEs, and severity of these events were assessed among chronic HCV patients receiving different combinations of DAAs in Pakistani Population. Such studies will lead us to generalize the data in terms of tolerability, evaluation of risk and safety profile of the local population.

## METHODS

This multicenter, analytical cross-sectional study was conducted at six gastroenterology and hepatology centers including Liver Center Faisalabad, Allama Iqbal Medical Institute and Liver Center DHQ Hospital Sialkot, Isra Hospital Hyderabad, Allied Hospital Faisalabad and Rehman Medical Institute Peshawar, between May 2018 and May 2019 after obtaining ethical approval from the PMA Ethics Committee (Reference Number: JA/758/LAE/67; Dated: 10^th^ April 2018). A total of 511 patients with chronic HCV receiving DAAs therapy were included in the study. Under primary inclusion, patients HCV of both genders with age > 18 years were considered while the patients with co-infection such as hepatitis B and/or human immunodeficiency virus (HIV), were kept under exclusion together with pregnant and/or lactating females. The patients were recruited using convenience sampling technique, data confidentiality was ensured as per ICH GCP requirements and written informed consent was taken from each patient prior to inclusion in the study.

The data was collected using a pre-designed questionnaire inquiring patient demographics, relevant history, concomitant medication and ongoing treatment plan recommended for chronic HCV. Moreover, the AEs associated with the prescribed treatment and its seriousness was also recorded as per the International Conference on Harmonization (ICH) classification and all reported AEs were presented by using the preferred term of Medical Dictionary for Regulatory Activities (MedDRA). The severity of medical events was assessed as per Karch and Lasagna classification^7^ which categorizes severity as minor when no antidote required nor therapy and/or prolongation of hospitalization needed, any change in drug therapy or any requirement of a specific treatment or hospitalization increased by at least one day was classified as moderate, whereas potentially life-threatening events, causing permanent damage or need of intensive medical care were classified under severe and lethal that directly or indirectly contributes to the death of the patient. Data on outcome assessment were based on actions taken due to AE, the outcome of the event (recovered or not), their suspected drugs as per physician observations along with the suspected dose.

The statistical analysis was carried out using SPSS version 20.0. Categorical variables were presented as frequencies & percentages while continuous variables were described as mean and standard deviation. Independent sample t-test was applied to assess the mean age of patients with developing AEs. The Chi-square test was applied to estimate the AE occurrence rate among HCV patients receiving Ribavirin-based treatment and those with cirrhosis, where p-value < 0.05 was considered as statistically significant.

## RESULTS

A total of 511 HCV patients receiving DAAs were included in the study of which 32% were males. Among the demographics, the mean age of the patients was found to be 43.5 ± 11.7 years, and the BMI was 25.3 ± 5.1 kg/m^2^. 87.3% of these patients were given DAAs in combination with weight-based ribavirin. The patient’s basic clinical and medical characteristics are given in Table-I.

**Table-I:** Basic clinical and medical characteristics of the enrolled patients (n=511).

Clinical and Medical Characteristics		*n*(%)
Patient History	Naive	435(85.1)
	Relapse	54(10.6)
	Non-Responder	22(4.3)
Cirrhosis	Non-cirrhotic	348(68.1)
	Compensated cirrhosis	43(8.4)
	Decompensated cirrhosis	53(10.4)
	Unknown	67(13.1)
Comorbidities	None	421(82.4)
	Diabetes Mellitus	44(8.6)
	Hypertension	57(11.2)
	CVD	6(1.1)
Others	Lichen Planus	1(0.2)
	Depression	3(0.5)
	Anxiety	2(0.4)
Treatment Approach	SOF, DAC and RBV	366(71.6)
	SOF and RBV	55(10.76)
	SOF and VLP	47(9.2)
	SOF and DAC	18(3.5)
	SOF, VLP and RBV	25(4.9)

*SOF: Sofosbuvir, RBV: Ribavirin, DAC: Daclatasvir, VLP: Velpatasvir

A total of 419 AEs were reported (considering multiple reported AEs), out of which majority (80.6%) patients experienced only one AE, and 15.3% were reported having two AEs after receiving HCV treatment regime. The most common among them was pyrexia (58.0%) followed by asthenia (11.2%) and fatigue (8.8%). Only 61 events were suspected from DAAs whereas the majority, 317 events were suspected with due to Ribavirin, five with concomitant therapy and remaining 36 were not classifiable. Out of these 61 AEs reported with DAAs, Sofosbuvir was administered in 54(88.6%) cases, Daclatasvir in 6(9.8%) and Velpatasvir was used in only 1(1.6%) case. Moreover, pyrexia, fatigue, headache, muscle spasm, asthenia, insomnia, palpitation, amnesia, abdominal pain upper, arthralgia, pain and vertigo were DAAs suspected AEs.

There was no serious AE reported as per the ICH classification. The severity distribution of AEs in 339 patients as per the Karch and Lasagna classification showed that 333(98.2%) patients were moderate inclusive of 325(97.6%) patients who received any corrective treatment, 4(1.2%) cases were of drug discontinuation, 3(0.9%) required both dose adjustment and appropriate treatment and only one had dose adjustment issue. The other 6(1.8%) were minor in nature as no further management was required.

The use of ribavirin-based treatments significantly affected the AE occurrence (p-value < 0.001). The study results illustrated that the AE occurrence was five times higher among HCV patients receiving ribavirin-based treatment and cirrhotic HCV patients were two times more likely to have potential AEs as compared to the non-cirrhotic HCV patients (Table-III).

**Table-II:** Adverse Events (AEs) as per MedDRA code among HCV patients taking medication.

Reported Adverse Events	*n*(%)
**Blood and lymphatic system disorders**	**14(3.3)**
Anaemia	14(3.3)
**Cardiac disorders**	**5(1.2)**
Palpitations	5(1.2)
**Eye disorders**	**1(0.2)**
Visual impairment	1(0.2)
**Gastrointestinal disorders**	**17(4.1)**
Abdominal discomfort	1(0.2)
Abdominal pain	2(0.5)
Abdominal pain upper	5(1.2)
Diarrhoea	2(0.5)
Gastroesophageal reflux disease	2(0.5)
Constipation	1(0.2)
Dyspepsia	3(0.7)
Vomiting	1(0.2)
**General disorders and administration site conditions**	**333(79.5)**
Pain	6(1.4)
Fatigue	37(8.8)
Pyrexia	243(58.0)
Asthenia	47(11.2)
**Immune system disorders**	**1(0.2)**
Asthma	1(0.2)
**Investigations**	**1(0.2)**
Weight decreased	1(0.2)
**Metabolism and nutrition disorders**	**1(0.2)**
Oedema peripheral	1(0.2)
**Musculoskeletal and connective tissue disorders**	**15(3.6)**
Arthralgia	3(0.7)
Muscle spasms	7(1.7)
Myalgia	2(0.5)
Pain in extremity	3(0.7)
**Nervous system disorders**	**19(4.5)**
Paraesthesia	2(0.5)
Dizziness	1(0.2)
Headache	11(2.6)
Amnesia	2(0.5)
Vertigo	3(0.7)
**Psychiatric disorders**	**8(1.9)**
Insomnia	7(1.7)
Depression	1(0.2)
**Renal and urinary disorders**	**1(0.2)**
Dysuria	1(0.2)
**Respiratory, thoracic and mediastinal disorders**	**1(0.2)**
Cough	1(0.2)
**Skin and subcutaneous tissue disorders**	**1(0.2)**
Pruritus	1(0.2)
**Vascular disorders**	**1(0.2)**
Epistaxis	1(0.2)

**Table-III:** Occurrence of Adverse Events in relation to the administration of Ribavirin-based treatment and cirrhosis.

Risk Factors	Adjusted OR*	95% CI	*p-value*
Ribavirin-based Treatment	5.21	2.30-9.10	<0.001
Cirrhosis Patient	1.90	1.10-3.12	0.019

## DISCUSSION

The safety and efficacy of DAAs based therapeutic regime for HCV infection has now been established.^8^-^10^ Nevertheless, the chronic HCV infections are known to intensify the renal deterioration and hence the safety, efficacy and tolerability among HCV patients with certain degrees of renal impairment remain poorly understood and require further investigation. In this multicenter cross-sectional study, we assessed the safety profile, occurrence and severity of AEs among the HCV patients receiving different combinations of DAAs. The current findings suggest that DAAs are generally well-tolerated among the Pakistani population with no serious AEs reported. A local Pakistani study from Lahore confirmed the effectiveness of Sofosbuvir in combination with Ribavirin among highly infected HCV patients, as 34 out of 35 patients actively achieved Sustained Virologic Response (SVR) with no major side-effects.^11^

Although being effective, it is essential to attain the real-world data verifying the safety and efficacy of this HCV treatment modality.^12^ Certain DAAs are not easily accessible due to high cost and hence its use might be limited in some states i.e. China restricts the use of DAAs as it is high-priced. However, adding to the real-world statistics, Zeng, Hu and their colleagues provided the safety and efficacy data concerning the use of different combination of DAAs for the treatment of HCV.^13^,^14^ In support the current study also established findings in favor of the safety of DAAs use i.e. among all the AEs experienced during HCV therapy with combinations of DAAs, there were no severe or lethal cases reported as per the severity classification. A recent pharmacovigilance study including the Egyptian population consuming Daclatasvir and Sofosbuvir in combinations with Ribavirin reported similar results displaying AEs in only 1.2% patients.^15^ Hence, the overall occurrence of AEs was greater in comparison to our reported rate i.e. 76% vs. 66%. Another study assessed the efficacy and safety of DAAs among the German population and reported a similar burden of AEs to be 63%.^16^

The most commonly reported events were found to be pyrexia and fatigue. Similarly, a local review highlighting the changing treatment modalities and DAAs dynamics among HCV patients reported that headache, fatigue and nausea were the most common side-effects associated with the use of Paritaprevir and Dasabuvir/Ombitasvir and Ritonavir, common DAA combination and only 1% of the total affected patients are reported with treatment discontinuation.^17^ The most commonly reported AEs among Chinese patients receiving DAAs were nausea, diarrhea, acid regurgitation and bilirubin elevation in some patients.^18^ Another study conducted among Argentinian patients showed anemia to be one of the most common AE among DAAs receiving patients. A study conducted in Rawalpindi, Pakistan reported that patients receiving DAAs experienced generalized weakness and fatigue in 27.1% of the patients and fever in 8.9% of the patients as the most common AEs.^19^

It was found that the Ribavirin-based therapy was found to be significantly associated with the occurrence of AEs. A recently published review article also endorses our findings. It maintains that compared to Ribavirin based therapies, DAAs have better safety profile.^6^ Ribavirin use has been significantly linked to anemia, especially among decompensated and End-Stage Renal Disease (ESRD) patients, therefore it use in addition to DAA is not recommendable as it may be harmful.^20^,^21^ Furthermore, the cirrhotic incidences added up to the occurrence of AEs among the enrolled HCV patients (p<0.05). It is evident that among HCV patients the presence of cirrhosis delays the treatment and negatively affects that therapeutic approach by significantly encouraging the occurrence of AEs.^17^ Additionally, the use of DAAs among HCV patients has been known to improve the glycemic control as per the results of a recent study conducted in Punjab.^22^ It was concluded that majority patients effectively achieved the optimal HbA1c levels and hence displayed improved Glycemic Control.^22^ Whereas, a local study has also provided data against the evident efficacy outcomes, suggesting that the Hepatocellular Carcinoma (HCC) was frequent among HCV patients treated with DAAs.^23^

### Limitations of the study

Although the study findings can be generalized to the whole population as it was multicenter including a large sample size but long-term follow-ups could enhance the knowledge regarding future implications of DAAs among the Pakistani population. Due to the design limitations, potential sources of bias can be information, sampling and interviewer bias and other limitation could be causality assessment of reported cases.

## CONCLUSION

DAAs are generally well-tolerated among the local Pakistani population as no serious AEs were reported. Among all AEs, experienced during HCV therapy, there was no severe or lethal cases reported as per the severity classification. The most commonly reported events with DAAs were pyrexia and fatigue. Additionally, HCV patients received Ribavirin with DAAs therapy as well as those who were cirrhotic had significant AEs risk. Hence it is recommended that such patients should be closely monitored.

### Authors Contribution:

**ZYH, MQZ, AB, MA, NA, MR, JBB:** Are responsible for the concept and study design.

**ZYH, MQZ, AB, MA, NA:** Contributed to the data collection and literature review.

**MR, JBB:** Are responsible for data analysis and interpretation and drafting of the manuscript.

**ZYH, MQZ, AB, MA, NA, MR, JBB:** Contributed to the critical review, revision and final approval of the study.

All the authors are equally responsible and accountable for the accuracy and integrity of the work.
